# Effect of circulating ceramides on adiposity and insulin resistance in patients with type 2 diabetes: An observational cross‐sectional study

**DOI:** 10.1002/edm2.418

**Published:** 2023-03-23

**Authors:** Emmanuel K. Ofori, Alfred Buabeng, Seth D. Amanquah, Kwabena O. Danquah, Seth K. Amponsah, Wormenor Dziedzorm, Francis K. Dogodzi, Laurinda X. Adusu‐Donkor, Segla K. Bernard, Henry Asare‐Anane

**Affiliations:** ^1^ Department of Chemical Pathology, U.G.M.S University of Ghana Accra Ghana; ^2^ Department of Clinical Pathology, NMIMR University of Ghana Accra Ghana; ^3^ Department of Medical Pharmacology, U.G.M.S University of Ghana Accra Ghana

**Keywords:** adiposity, ceramide, insulin resistance

## Abstract

**Introduction:**

Insulin resistance (IR) is one of the common chronic metabolic disorders in Africa and elsewhere. Accumulation of lipids in the body may be due to an imbalance in the metabolism of lipids, glucose and proteins. Ceramides are a sphingolipid class of lipids that are biologically active and vital in the production of more complex lipids. Circulating ceramides are thought to have a role in the development of obesity‐related IR, although the precise involvement remains unclear.

**Aim:**

To investigate the impact of circulating ceramide on IR and body adiposity in people with and without type 2 diabetes mellitus (T2DM).

**Methodology:**

The study was observational and cross‐sectional. There were a total of 84 volunteers with T2DM and 75 nondiabetics (control). The participants' ages, body mass indexes (BMI), waist circumferences, and blood pressure (BP) were among the clinical parameters assessed. Ceramide levels, fasting plasma glucose (FPG), lipids, basal insulin levels and glycated haemoglobin (HbA1c) were also measured. Additionally, the homeostatic model assessment for IR (HOMA‐IR) and beta cell function (HOMA‐β) were computed.

**Results:**

T2DM and control participants had different mean values for anthropometric parameters, BP, FPG, HbA1c, lipids, insulin, HOMA‐IR, HOMA‐β and ceramide levels (*p* < .05 for all). HOMA‐IR, HOMA‐β and cardiovascular risk were significant correlates with ceramide levels in the T2DM group (r = 0.24; −0.34; 0.24, *p* < .05, respectively). Further, FPG (OR = 1.83, *p* = .01) and ceramide (OR = 1.05, *p* = .01) levels were significant predictors of IR in the case group.

**Conclusion:**

Patients with T2DM exhibited high ceramide concentrations, which, when combined with high FPG, were associated with IR. The consequences of circulating ceramides in health and disease; however, merit further research.

## Abstract 2

The exact role of circulating ceramides in obesity‐related insulin resistance remains elusive. In this study patients with type 2 diabetes exhibited higher ceramide concentrations, which, when combined with elevations in blood glucose, were associated with insulin resistance.

## 1 INTRODUCTION

Insulin resistance (IR) is characterized by diminished responsiveness of peripheral tissues to the action of insulin; often leading to hyperglycemia. The aforementioned can lead to a reduction in postprandial glucose storage, particularly in the skeletal muscle and liver.^1^, ^2^, ^3^ IR is known to affect about 15.5%–46.5% of the human population.^4^, ^5^ A sedentary lifestyle, heredity, a high‐fat diet, obesity, type 2 diabetes mellitus (T2DM) and heart‐related pathologies have all been associated with IR.^6^, ^7^, ^8^ Indeed, obesity‐mediated IR is common in individuals who live in areas defined by persistent energy surplus, a low basal metabolic rate or a combination of both.^9^, ^10^, ^11^ Individuals affected by these metabolic derailments often have vision‐related challenges, have their lower limbs amputated and had kidney failure, among others.^12^, ^13^, ^14^

There is still a paucity of information as to how obesity can contribute to IR and T2DM. One hypothesis being explored is that when lipids buildup more than their oxidative demands, they spill over into detrimental metabolic pathways.^15^, ^16^, ^17^ Ceramides are essential components of bioactive lipids that are produced when sphingomyelin, sphingosine or fatty acids are hydrolysed.^18^, ^19^, ^20^ The amount of ceramide that is produced can be controlled by the presence of long‐chain saturated fatty acids in the endoplasmic reticulum. These fatty acids are involved in the process of de novo ceramide synthesis.^21^, ^22^ Ceramide levels have been associated with glucose metabolism dysregulation^23^, ^24^ and subsequent IR in humans, albeit the precise mechanism is unknown. Reducing peripheral glucose absorption, inactivating Akt and generating an inflammatory milieu (activating tumour necrosis factor‐alpha) are all conceivable mechanisms that have been proposed for how a high concentration of ceramides could cause IR.^25^, ^26^, ^27^

In sub‐Saharan Africa, there is very little information or data on the role of circulating ceramides and IR. Currently, available treatment options for IR and T2DM focus on lowering cholesterol and triglycerides. However, IR can occur even when cholesterol and triglyceride levels are normal, which suggests that other possible lipids and their intermediates are involved in the process. Knowing basal ceramide levels may have potential implications in the clinical management of patients with metabolic disorders especially in developing countries and in limited‐resource settings. The study's goal was to look into the effect of circulatory ceramide on IR and body adiposity in persons with T2DM. We hypothesize that increasing levels of circulating ceramide will be related to an increase in IR and body adiposity among research participants.

## 2 MATERIALS AND METHODS

### 2.1 Study design, site and participants

This was an observational cross‐sectional research. One hundred and fifty‐nine (159) volunteers were recruited from the St. Gregory Catholic Hospital (SGCH), which is located in Gomoa Buduburam, Central Region, Ghana. Of the participants, 84 were diagnosed with T2DM, while 75 did not have DM, and served as control. Participants were to be 18 years and above. The control group had no history of T2DM and had fasting plasma glucose (FPG) levels that were lower than 5.6 mmol/L. Further, our control group had glycated haemoglobin (HbA1c) levels between 4% and 5.6%, and thus met the criteria in the classification of a nondiabetic.^28^ Patients who had been diagnosed with tuberculosis, immunocompromised people, pregnant and nursing mothers, patients undergoing therapy for COVID‐19 and persons who were receiving insulin for the treatment of DM were not included in the research. The Ethical and Protocol Review Committee (EPRC) of the College of Health Sciences at the University of Ghana approved this research (ID: CHS‐EtM.2‐4.4/2020–2021). Additionally, permission was requested from the Administration of St. Gregory Catholic Hospital (SGCH). Before recruitment, participants were required to complete an informed consent form indicating that they were willing to take part in the study. The participants were given information on the following when they signed their consent forms: the rationale and duration of the study, the benefit of the study, the materials used in sample collection and the possible dangers of the sampling procedure. It was requested of the participants that they comply with all COVID‐19 protocols. A T2DM prevalence rate of 6.46% was used in the calculation of the sample size.^29^

### 2.2 Clinical assessment and laboratory procedures

To obtain sociodemographic and clinical information from research participants, a standard questionnaire was employed in conjunction with a review of patients' folders. The questionnaire obtained information about the participants' names, ages, gender, marital status, living styles, family history of DM and times of onset of DM. The weights of the participants were measured by using a weighing scale (Omron, USA). A wall‐mounted stadiometer was used to measure the height of subjects. Body weight and height measured were to 1.0 kg and 0.005 m of their approximate measurements, respectively. Participants wore little clothing and were barefoot when their heights and weights were measured. Body mass index (BMI) was determined by dividing weight by the square of height (Kg/m^2^).

BMI was further used to categorize patients into obese 1 (30–34.9 Kg/m^2^), obese 2 (35–39.9 Kg/m^2^) and obese 3 (≥40 Kg/m^2^).^30^ Using a tape measure, standing waist circumference (WC) to the nearest 0.1 cm around the navel or at the level of the umbilicus was measured. In addition, blood pressure (BP) was recorded after 10–15 min of rest while the volunteer sat in a chair. The BP was measured using an automated cuff BP machine. A validated formula was used to estimate the body fat percentage. For men, the formula was 0.567 × WC + 0.101 × age (yrs.) − 31.8, while for females, the formula was 0.438 × WC + 0.221 × age (yrs.) − 9.4.^31^

Before blood sample collection, the participants were instructed to abstain from food for 10–14 h overnight. A normal blood draw of 5 mL was performed on each participant while they were fasting by a qualified phlebotomist during the hours of 7:00–9:00 AM. Quantification of glycosylated haemoglobin (HbA1c) and fasting plasma glucose (FBP) was performed using 1 mL of the blood sample that was in an EDTA tube and another 1 mL of blood that was in a fluoride oxalate tube, respectively. To obtain sera, the remaining 3 mL were transferred to a serum separator tube, which was then put on a rack at a temperature of 25°C for 10–20 min before being centrifuged at 3000 rpm for 10 min. The resulting sera were divided into 0.5 mL portions and placed in Eppendorf tubes. These were then kept at a temperature of −20°C for about a month until analysis. The sandwich ELISA approach (GenWay Biotech Inc) was used to determine the ceramide and insulin concentrations present in sera. The test involved the engagement of a double‐specific monoclonal antibody to provide accurate results. Fluorescence immunoassay (ichorma™) was used to determine the HbA1c levels in the blood (Boditech Med Inc). An automated chemical analyser was used to obtain concentrations of FPG and lipids (Mindray BS430, China). The homeostatic assessment for IR (HOMA‐IR) and beta cell function (HOMA‐β) were computed employing a formula that has been previously described.^32^, ^33^

### 2.3 Statistical analysis

A Microsoft Excel (2010) spreadsheet was used to compile all data. Statistical analysis was done using Statistical Package for the Social Sciences (SPSS version 22.0). Data were presented as descriptive statistics and summarized in tables for presentation. The independent samples t‐test, Pearson's product–moment correlation coefficient (*r*) and a logistic regression model were used to investigate the influence of anthropometry, clinical and biochemical characteristics on selected parameters. A *p*‐value of <.05 was considered statistically significant.

## 3 RESULTS

The study had a total of 149 participants, including 84 with T2DM (the case group) and 75 as controls. In the case group, there were 68 females, making up 81% of the total. In the control group, there were 47 females, making up 62.7% of the total. The majority (42%) of the cases had completed basic education in contrast to the control group where almost all (90.7%) had completed tertiary education. Moreover, about a third (21.4%) in the case group were insulin‐sensitive (Table 1).

**TABLE 1:** Sociodemographic and clinical (categorical) characteristics of study participants.

Features	Case group	Control group
	Number (%)	Number (%)
Gender		
Male	16 (19.0)	28 (37.3)
Female	68 (81.0)	47 (62.7)
Education level		
None	15 (17.9)	0 (0.0)
Basic	39 (46.4)	1 (1.3)
Secondary	20 (23.8)	6 (8.0)
Tertiary	10 (11.9)	68 (90.7)
Body mass index		
Normal	13 (15.5)	25 (33.3)
Underweight	2 (2.4)	2 (2.7)
Overweight	31 (36.9)	24 (32,0)
Obese 1	21 (25.0)	17 (22.7)
Obese 2	13 (15.5)	5 (6.7)
Obese 3	4 (4.8)	2 (2.7)
Insulin status		
Sensitive	18 (21.4)	38 (50.7)
Resistant	66 (78.6)	37 (49.3)

*Note*: Participant demographics and clinical data (categorical variables) are shown in the table. Data are presented as frequencies and percentages in brackets. BMI categories included obese 1 (30–34.9 Kg/m^2^), obese 2 (35–39.9 Kg/m^2^) and obese 3 (≥ 40 Kg/m^2^).

The clinical (continuous), anthropometric and biochemical information of the study population is displayed in Table 2. The average age of the participants in the case group was 53.7 ± 9.5 years, whereas the average age of the volunteers in the control group was 31.9 ± 6.5 years. The case group had higher WC, waist‐to‐hip ratios, BMI, percentages of body fat, blood pressure (both systolic and diastolic), ceramide levels, FPG, total cholesterol, triglycerides, LDL‐cholesterol and HbA1c levels than the control group (*p* < .05 for all). On the contrary, insulin levels and HOMA‐β were higher in the control group compared with the case group (*p* < .05 for both). HOMA‐IR levels were, however, comparable between the cases and controls (2.91 ± 1.35 vs. 2.55 ± 1.54; *p* = .092).

**TABLE 2:** Clinical (continuous) and biochemical characteristics of the study participants.

Features	Diabetes group	Control group	*p*‐value	95% CI
	Mean ± SD	Mean ± SD		
Age (years)	53.7 ± 9.4	31.8 ± 6.4	<.001	19.2–24.4
BMI (kg/m^2^)	30.1 ± 6.0	27.6 ± 5.8	.013	0.6–4.3
Duration of DM (years)	6.1 ± 5.0	—		
Hip circumference (cm)	109.1 ± 12.3	106.8 ± 13.2	.251	−1.6–6.5
Waist‐to‐hip ratio	0.9 ± 0.1	0.8 ± 0.1	<.001	0.1–0.2
Waist‐to‐height ratio	0.7 ± 0.1	0.5 ± 0.1	<.001	0.1–0.3
Percent body fat (%)	43.2 ± 9.6	31.2 ± 11.0	<.001	8.8–15.3
Systolic BP (mmHg)	132.6 ± 18.3	113.2 ± 10.3	<.001	14.8–23.9
Diastolic BP (mmHg)	81.8 ± 11.5	71.5 ± 8.9	<.001	7.1–13.5
FBG (mmol/L)	11.1 ± 5.3	4.8 ± 0.8	<.001	5.1–7.5
HbA1c (%)	7.2 ± 2.8	4.2 ± 0.5	<.001	2.8–3.7
Total cholesterol (mmol/L)	5.9 ± 1.3	5.0 ± 1.2	<.001	0.5–1.3
Triglycerides (mmol/L)	1.3 ± 0.5	0.9 ± 0.3	<.001	0.3–0.8
HDL‐C (mmol/L)	1.5 ± 0.4	1.2 ± 0.3	<.001	0.1–0.4
LDL‐C (mmol/L)	3.8 ± 1.1	3.3 ± 1.1	.010	0.1–0.8
Insulin (mIU/L)	6.3 ± 2.5	11.7 ± 9.5	<.001	−7.1–3.7
HOMA‐IR	2.91 ± 1.35	2.55 ± 1.54	.092	0.09–0.88
HOMA‐β	32.8 ± 32.5	208.1 ± 185.5	<.001	−218.5–134.0
Ceramide (pg/mL)	80.8 ± 22.3	64.1 ± 27.6	<.001	8.8–24.6
Cardiovascular risk	4.2 ± 1.2	4.3 ± 1.0	.660	−0.4–0.3

Abbreviations: BMI, body mass index; BP, blood pressure; FPG, fasting plasma glucose; HbA1c, glycated haemoglobin; HDL‐C, high‐density lipoprotein cholesterol; HOMA‐IR, the homeostatic model assessment of insulin resistance; HOMA‐β, the homeostatic model assessment of beta cell function; LDL‐C, low‐density lipoprotein cholesterol. In this table, the clinical (continuous) and biochemical features of study participants are outlined. The data are provided as means and standard deviations, with a significance threshold of 0.05.

The information contained in Table 3 provides an overview of the associations that exist between the levels of ceramide and the levels of the other clinical and biochemical markers in individuals who participated in the study. The levels of ceramide in the case population were found to be associated with HOMA‐IR, HOMA‐β and the risk of cardiovascular disease (*r* = 0.24, −0.34 and 0.24; *p* < .05 for all). There was, however, no association between ceramide levels versus BP and lipid profile parameters in the case group (*p* > .05 for all). In the control group, HOMA‐IR, HOMA‐β, BMI, waist‐to‐hip ratio, insulin, % body fat, waist circumference, hip circumference and waist‐to‐height ratio were all associated with ceramide levels (*r* = 0.33, −0.26, 0.37, 0.28, 0.32, 0.28, 0.42, 0.27 and 0.43; *p* < .05 for all). When compared with the other variables evaluated as potential predictors of IR, FPG (odds ratio = 1.83, *p* = .01) and serum ceramides (odds ratio = 1.05, *p* = .01) were found to be significant predictors of IR among the case‐cohort (about two and one‐fold higher potential predictability risks, respectively) (Table 4).

**TABLE 3:** Relationship between levels of ceramide and clinical/biochemical correlates.

Parameters	Diabetes group			Control group	
	*r*	*p*		*r*	*p*
Total cholesterol	−.01	.990		−.13	.290
Triglycerides	−.06	.570		−.02	.970
HDL‐C	−.02	.890		−.12	.320
LDL‐C	.05	.660		−.16	.160
Insulin	.04	.730		.32	.007
HOMA‐β	−.34	.003		−.26	.030
Systolic BP	−.08	.450		−.05	.680
Diastolic BP	.09	.410		−.08	.510
Waist‐to‐hip ratio	−.10	.370		.28	.020
FPG	.17	.120		.18	.120
HbA1c	.06	.620		.08	.490
HOMA‐IR	.24	.041		.33	.004
AIP	.23	.060		.18	.120
Cardiovascular risk	.24	.030		.06	.630
BMI	.15	.180		.37	.001
% body fat	.02	.970		.28	.030
Waist circumference	.10	.360		.42	.001
Hip circumference	.18	.102		.27	.020
Waist‐to‐height ratio	.16	.140		.43	.001

Abbreviations: AIP, an atherogenic index of plasma; BMI, body mass index; BP, blood pressure; FPG, fasting plasma glucose; HbA1c, glycated haemoglobin; HDL‐C, high‐density lipoprotein cholesterol; HOMA‐IR, the homeostatic model assessment of insulin resistance; HOMA‐β, the homeostatic model assessment of beta cell function; LDL‐C, low‐density lipoprotein cholesterol. In this table shows the relationship between levels of ceramide and several clinical and biochemical correlates of the study participants. r is the correlation coefficient. *p*‐value was considered significant at .05 alpha level.

**TABLE 4:** Predictors of insulin resistance among individuals in the case‐cohort.

Predictors	Beta	SE	Wald	*OR*	*p‐*value	95% CI
Duration of diabetes	−0.01	0.07	0.01	0.99	.91	0.87–1.14
Systolic BP	0.03	0.03	1.03	1.03	.31	0.97–1.09
Diastolic BP	0.07	0.05	2.09	1.07	.15	0.98–1.17
BMI	−0.21	0.11	3.49	0.84	.05	0.65–1.01
FPG	0.61	0.24	6.60	1.83*	.01	1.15–2.91
HbA1c	−0.41	0.23	3.22	0.66	.07	0.42–1.04
HDL‐C	0.60	2.02	0.09	1.81	.77	0.04–95.05
LDL‐C	−0.65	0.76	0.73	0.52	.39	0.12–2.32
Ceramide	0.05	0.02	6.45	1.05*	.01	1.01–1.10
Cardiovascular risk	1.34	1.13	1.43	3.83	.23	0.42–34.74
% body fat	0.05	0.05	0.71	1.05	.20	0.94–1.16
Hip circumference	0.05	0.06	0.67	1.05	.41	0.94–1.17
HOMA‐β	0.03	0.02	1.49	1.03	.22	0.99–1.07

Abbreviations: BMI, body mass index; BP, blood pressure; FPG, fasting plasma glucose; HbA1c, glycated haemoglobin; HDL‐C, high‐density lipoprotein cholesterol; HOMA‐β, homeostatic model assessment of beta cell function; LDL‐C, low‐density lipoprotein cholesterol; OR, odds ratio; SE, Standard error. In this presents the factors that can be used to predict IR in the diabetic group.

* Significant at the 0.05 alpha level.

## 4 DISCUSSION

The primary objective of this study was to determine whether or not individuals in Ghana with T2DM who were obese had altered levels of circulatory ceramides. It is well known that the capacity of sphingolipids to regulate cellular activity is of importance in several bodily processes (physiological and pathological). In this study, participants with TDM had higher serum ceramide levels than their nondiabetic counterparts. Broskey and colleagues earlier reported that subjects who were both obese and diabetic had higher levels of ceramides in their skeletal muscle, while subjects who were neither obese nor diabetic had significantly lower levels of ceramides in their skeletal muscle.^34^ Indeed, the plasma concentration of dihydroceramide, a precursor to ceramide, has been linked to an increased likelihood of developing diabetes in mice and humans.^35^ Within our case population, the metrics of insulin resistance, insulin sensitivity and cardiovascular risk all demonstrated significant relationships with ceramide levels and agreed with prior studies^36^ (Table 3). In both human and animal models, ceramide levels in the blood, adipose tissue, the liver and skeletal muscle are inversely correlated with the degree of insulin sensitivity.^36^, ^37^, ^38^

Although not observed in the present study, a recent study lends support to the observation that ceramide accumulation plays a role in sarcopenia that is detrimental to the body.^39^ The authors concluded that as people get older, ceramides begin to buildup in the muscle, hence affecting the function of the muscle. Yet, older persons who had a genetic mechanism that reduced ceramide production in their muscle tissues were fitter than expected for their age, evidenced by greater handgrip strength, as well as the capacity to walk long distances and stand up from a chair. Thus, ceramides in muscle tissues and other parts of the body may be the mediators of multiple processes in cells. Ageing skeletal muscle has been associated with greater muscle ceramides in some studies^40^, ^41^ but not in others^42^, ^43^ with these variations partly being explained by BMI status and physical activity levels, given that ceramide is linked to insulin resistance in individuals with increased BMI. A positive connection was also identified between age and a panel of plasma ceramides in a cohort consisting of 164 patients (84 women) ranging in age from 19 to 80 years old.^44^ When the results were analysed according to the menopausal status of the participants, it was discovered that the levels of long‐chain ceramides, very long‐chain ceramides and dihydroceramide were higher in postmenopausal women than they were in premenopausal women. The same study found a substantial inverse association between the circulating levels of estradiol and ceramide (d18:1/24:1) in women of all ages. This correlation was not found in men, which suggests that estradiol may regulate the circulating levels of ceramide in a sexually dimorphic manner.

Ceramides have also been reported to vary with oestrogen levels. In their study of older female adults, Kendall and colleagues recently discovered a correlation between advancing age, elevated oestrogen levels, and changes in the quantity and length of ceramides. As part of a targeted lipidomics investigation, liquid chromatography–tandem mass spectrometry was utilized to investigate ceramides and several other sphingolipids. In comparison with the premenopausal group, postmenopausal women had lower levels of serum oestrogen and a lower abundance of stratum corneum ceramides. Different ceramide species from different ceramide classes showed significant reductions in postmenopausal women; however, these reductions were not observed in postmenopausal women who were using hormone replacement therapy (HRT). In addition, the treatment of primary human keratinocytes with estradiol (10 nM) led to an increase in the formation of ceramides, which suggests that oestrogen plays a direct regulatory role in the metabolism of keratinocyte ceramides.^45^ Another in vitro study conducted with human cancer cells that expressed oestrogen receptors showed that a considerable reduction in ceramide buildup could be achieved through incubation with estradiol.^44^ Taken together, these findings show that ageing is linked to levels of circulating ceramide, which, in postmenopausal women, appear to be regulated by levels of estradiol.

Additionally, BMI, waist‐to‐height ratio, insulin resistance, adiposity, percent body fat, waist circumference and waist‐to‐hip ratio were all significant correlates in the control group and were consistent with those found in previous research.^46^ An overabundance of fatty acids can lead to an increase in tissue ceramide formation, perhaps as a result of sphingolipid recycling or actions along the salvage pathway. It has been demonstrated that an increase in the concentration of free fatty acids in plasma leads to an increase in the activity of enzymes involved in the metabolism of ceramides, especially serine palmitoyl transferase (SPT), which in turn promotes the production of de novo ceramides.^47^ Indeed, people who are overweight in general or obese limited to the abdominal region have been found to have higher levels of ceramides in their muscles, which has been linked to insulin resistance in those muscles.^48^, ^49^ Due in part to an increase in the influx of fatty acids and a buildup of ceramides, obesity causes the dysregulation of various cell‐intrinsic pathways, which in turn impairs the transmission of insulin signalling molecules.^50^ Furthermore, data suggest that an accumulation of ceramide subtypes in the tissues of insulin‐resistant animals and humans inhibits or impairs insulin action and glucose uptake by inactivating the protein kinase B pathway^51^ and the glucose transporter type 4.^52^ Therefore, ceramides prevent glucose from being absorbed and hinder the storage of glycogen and triglycerides.^53^ This results in elevated amounts of glucose in the blood as well as poor glycaemic control. Reports suggest that blocking ceramide biosynthesis and/or catalysing ceramide breakdown in humans and rodents ameliorates numerous metabolic diseases including diabetes mellitus, cardiovascular disease and steatohepatitis^20^, ^54^ hinting at a direct relationship. The deleterious effects of ceramides have also been shown in muscles during Duchenne disease.^55^

Insulin sensitivity (HOMA‐β) was found to be associated with a reduction in total ceramide concentration and agreed with the findings of a prior study denoted as the strong heart family study (SHFS). The study discovered various ceramide species that were related to fasting insulin levels, HOMA‐IR levels and HOMA‐B levels both at the baseline and follow‐up.^56^ Our results further complement another study that was conducted on obese participants who had undergone bariatric surgery (Hazel et al., 2011). The study had 13 diabetic patients who were obese and had undergone surgery to have their stomachs bypassed using a gastric sleeve as text subjects. The levels of ceramide subspecies, as well as selected biochemical parameters, were determined before surgery, three months after surgery and six months post‐surgery. There was a considerable reduction in fasting lipids, as well as an increase in insulin sensitivity and HDL cholesterol levels, 3 months following surgery compared with presurgical values. Following surgery, a decrease in total ceramide levels and an increase in insulin sensitivity were among the changes observed, indicating a bidirectional relationship between total ceramide concentration and insulin sensitivity (Hazel et al., 2011). Furthermore, ceramides and IR are strongly linked. In addition to transporting ‘bad’ cholesterol in the blood, LDL is responsible for moving ceramides around the body. Ceramides promote LDL entry into vessel walls, which in turn encourages plaque development. The existence of a positive connection between LDL‐ceramide and insulin resistance (HOMA‐IR) lends weight to the notion that LDL‐ceramide plays a pathogenic role. In a separate but related study, circulating LDL‐ceramides led to IR and inflammation in lean mice, and they decreased glucose disposal uptake in cultured myotubes.^37^ Ceramides, thus, contribute to the homeostatic control of lipid metabolism by influencing the absorption, storage and oxidation of fatty acids in adipocytes, hepatocytes and myocytes.^57^, ^58^

Blood pressure, BMI, percent body fat, FPG, HbA1c, total cholesterol, triglycerides and low‐density lipoproteins were significantly higher among the case group when compared with the control group. This finding corroborates other studies.^59^, ^60^ Chronic caloric excess, fat composition and distribution variability, may play a role in the development and increased prevalence of insulin resistance and cardiometabolic disturbances.^61^, ^62^

Additionally, we sought to find out the factors that are diagnostic of IR in our case‐cohort. Only FPG levels and ceramide levels were demonstrated to be predictive of IR in the T2DM group. Specifically, the likelihood of our case group being insulin resistant increased by nearly twofold when elevated levels of FPG were present and by onefold when elevated levels of ceramides were present. These findings are in line with what others have reported in the past.^17^, ^34^, ^63^

Although a correlation between TG/HDL ratio and IR has been well documented in the context of major clinical studies, the present investigation could not find a meaningful connection between the two. For example, a higher triglyceride to high‐density lipoprotein cholesterol ratio (TG/HDL) was found to be an independent predictive factor and positively linked with an increased risk of incident diabetes in a recent large retrospective study that was conducted on Chinese males who were at least 20 years old.^64^ Another large cross‐sectional study, with 1608 nonobese middle‐aged and elderly individuals, found that the TG/HDL‐C ratio was the best marker for predicting IR in the nonobese subjects, with the area under the receiver operating characteristic curve (AUC) of 0.73, *p* < .001. Increased TG/HDL‐C ratio was related to IR and cell function in Hispanic and African American men and women, similarly to Caucasians, and could predict the incidence of type 2 diabetes in women.^65^

It would appear that this is the first time that the levels of ceramide in blood have been reported in the sub‐Saharan region. Until now, there has been no known research conducted in Ghana that examines the relationship between elevated levels of circulating ceramide and increased risk of IR and obesity. The findings of this study may serve as a baseline for subsequent research in African communities.

This study had some limitations. Given that this was a cross‐sectional study, we were limited in our ability to identify the underlying causes of the associations found. Follow‐up information would be invaluable in determining whether or not our findings are the result of causal linkages. Other factors, such as diet and level of physical activity, were not taken into account in this study. Further, the authors could only detect total ceramide concentrations in the blood. This study might have benefited from measurements of specific ceramide subspecies, which could have revealed which subspecies are the most harmful and related to IR. The measurement of serine palmitoyl transferase activity, oestrogen levels and ceramide levels contained in LDL would have bolstered the findings from this study. A hyperinsulinaemic‐euglycaemic clamp procedure to determine glucose disposal rates would have been ideal for this study since increased basal insulin levels may not necessarily depict an abnormality.

## 5 CONCLUSION

T2DM participants displayed noticeably higher serum ceramide concentrations, which together with elevated FPG levels were significant predictors of IR. This finding gives credence to the fact that ceramide metabolism is altered in individuals with IR, and that an excess of ceramide can be harmful to metabolic health.

## PARTICIPANT CONSENT STATEMENT

All participants provided written informed consent.

## CLINICAL TRIAL REGISTRATION

The study protocol has not been registered as a clinical trial.

## AUTHOR CONTRIBUTIONS

**Emmanuel Kwaku Ofori:** Conceptualization (lead); funding acquisition (equal); resources (equal); supervision (lead); writing – original draft (lead); writing – review and editing (equal). **Alfred Buabeng:** Data curation (lead); formal analysis (lead); methodology (equal); resources (equal); writing – original draft (supporting). **Seth Amanquah:** Supervision (supporting); writing – review and editing (supporting). **Kwabena Owusu Danquah:** Funding acquisition (supporting); resources (supporting); writing – review and editing (supporting). **Seth K Amponsah:** Resources (supporting); writing – review and editing (supporting). **Wormenor Dziedzorm:** Data curation (supporting); formal analysis (supporting); writing – review and editing (supporting). **Francis K Dogodzi:** Data curation (supporting); investigation (equal); writing – review and editing (supporting). **Laurinda Xolali Adusu‐Donkor:** Data curation (supporting); writing – review and editing (supporting). **Bernard Kwame Segla:** Data curation (supporting); writing – review and editing (supporting). **Henry Asare‐Anane:** Supervision (supporting); writing – review and editing (supporting).

## FUNDING INFORMATION

Emmanuel Kwaku Ofori and Kwabena Owusu Danquah obtained a Seed Grant from BANGA‐Africa (UG‐BA/SRG‐001/2021).

## CONFLICT OF INTEREST STATEMENT

The authors declare no competing financial interests.

## ETHICAL APPROVAL STATEMENT

The Ethical and Protocol Review Committee (EPRC) of the College of Health Sciences at the University of Ghana approved this research (ID: CHS‐EtM.2‐4.4/2020–2021). Additionally, permission was requested from the Administration of St. Gregory Catholic Hospital (SGCH).

## Data Availability

The corresponding author will provide the interested party with access to the datasets that were used throughout this study upon reasonable request.
